# Comparison of different artificial intelligence tools’ answers to questions related to early intervention: ChatGPT versus Gemini

**DOI:** 10.1590/1806-9282.20251434

**Published:** 2026-06-19

**Authors:** Ayşegül Yildirim, Aslıhan Turan, Hasan Gerçek, Gülhan Çaka

**Affiliations:** 1 KTO Karatay University, Vocational School of Health Sciences, Department of Child Development – Konya, Turkey.; 2 KTO Karatay University, Faculty of Health Sciences, Department of Midwifery – Konya, Turkey.; 3 Acıbadem University, Faculty of Health Sciences, Department of Physical Therapy and Rehabilitation, Istanbul, Turkey.; 4 Mersin Bozyazı State Hospital, Department of Pediatric – Mersin, Turkey.

**Keywords:** Artificial intelligence, Early intervention, Health communication, Readability

## Abstract

**OBJECTIVE:**

The aim of this study was to assess the quality and readability of ChatGPT and Gemini's responses to frequently asked questions about early intervention for individuals with at-risk infants.

**METHODS:**

Ten frequently asked questions about early intervention were selected by three researchers (a child development specialist, a physiotherapist, and a midwife) from a list generated by ChatGPT and Gemini. Questions were sent to ChatGPT version 4.0 and Gemini 1.5, and initial responses were recorded without follow-up queries. Ten independent experts (two special education specialists, two child development specialists, two physiotherapists, two midwives, and two pediatricians) The quality of ChatGPT and Gemini's responses was assessed using a four-grade rating system. Readability levels were analyzed using the Flesch-Kincaid Grade Level through WordCalc software.

**RESULTS:**

One of the answers given by ChatGPT was of higher quality than Gemini (p=0.025), while one answer given by Gemini was of higher quality than ChatGPT (p=0.033). The answers to the other questions were of similar quality, with Gemini having a lower level.

**CONCLUSION:**

This study compares the quality and readability of the answers given by artificial intelligence-based language models to demonstrate their potential to appeal to different user groups. While the models generally provided answers of similar quality, quantitative differences in readability were observed, suggesting potential suitability for different audiences. These findings contribute to understanding the role of AI tools in health communication.

## INTRODUCTION

Artificial intelligence technology has experienced very rapid developments in recent years, and this has become a part of daily life by affecting studies in communication, entertainment, education, health, and many other fields^1,2^. These rapid developments in the field of artificial intelligence (AI) have led to different AI models^3^. Large language models (LLMs), as AI models, are AI systems trained on countless words generated from books, articles, and internet-based content^4^.

The most popular is ChatGPT, a language modeling tool developed by OpenAI that allows people to chat with a machine on different topics, generating responses to user-specified prompts^5,6^. ChatGPT is a generative AI chatbot, and other AI models are being developed through similar processes^4^.

Gemini is another powerful language model. Recognizing the power and impact of AI, Google DeepMind has ­developed Gemini, an intelligent chat tool with advanced capabilities. Gemini gives users access to accurate and relevant answers by utilizing the database accumulated by Google^7^. Gemini is trained not only on text data but also on various types of data, such as audio, image, and video, so it works by processing integrated data types^8^.

ChatGPT is leading the way in tailoring personalized mental health support and improving chronic condition management. Gemini advanced offers support in facilitating early detection of diseases and medical decision-making^9^. People can use AI models when searching for health-related information^10^. Parents use online resources to learn about their baby's development and health^11,12^.

At-risk infants are characterized by adverse environmental and biological factors that occur during pregnancy, at birth, or after birth and contribute to the development of neurodevelopmental disorders and increased mortality^13^. Parents who have at-risk babies may have problems in terms of travelling to the health institution, transportation, cost, time, etc. With the development of AI models, it can be a good support for parents to guide and help their children^14^.

According to a study conducted at the University of Kansas, parents seeking information about their children's health trusted AI more than health professionals, and evaluated a text generated by AI as reliable and credible^15^. Given the rapid development and increasing popularity of AI and its impact on the dissemination of health-related information, it is extremely important to assess the accuracy and quality of ChatGPT and Gemini^16^. To the best of our knowledge, no study has investigated the questions of parents with at-risk infants in AI models. The aim of this study is to compare the quality and readability of ChatGPT and Gemini's answers to the questions asked by individuals with high-risk babies about early intervention.

## METHODS

### Question collection process

The purpose of using ChatGPT and Gemini to generate an initial pool of questions was not to identify empirically validated "frequently asked questions," but rather to capture how LLMs conceptualize common parental concerns regarding early intervention. This approach was chosen deliberately to align with the study's primary aim: To compare the responses generated by AI systems to questions formulated within their own knowledge frameworks. Therefore, the generated questions reflect the models’ internal representations of commonly asked questions rather than real-world parental query frequencies.

**Figure 1:**
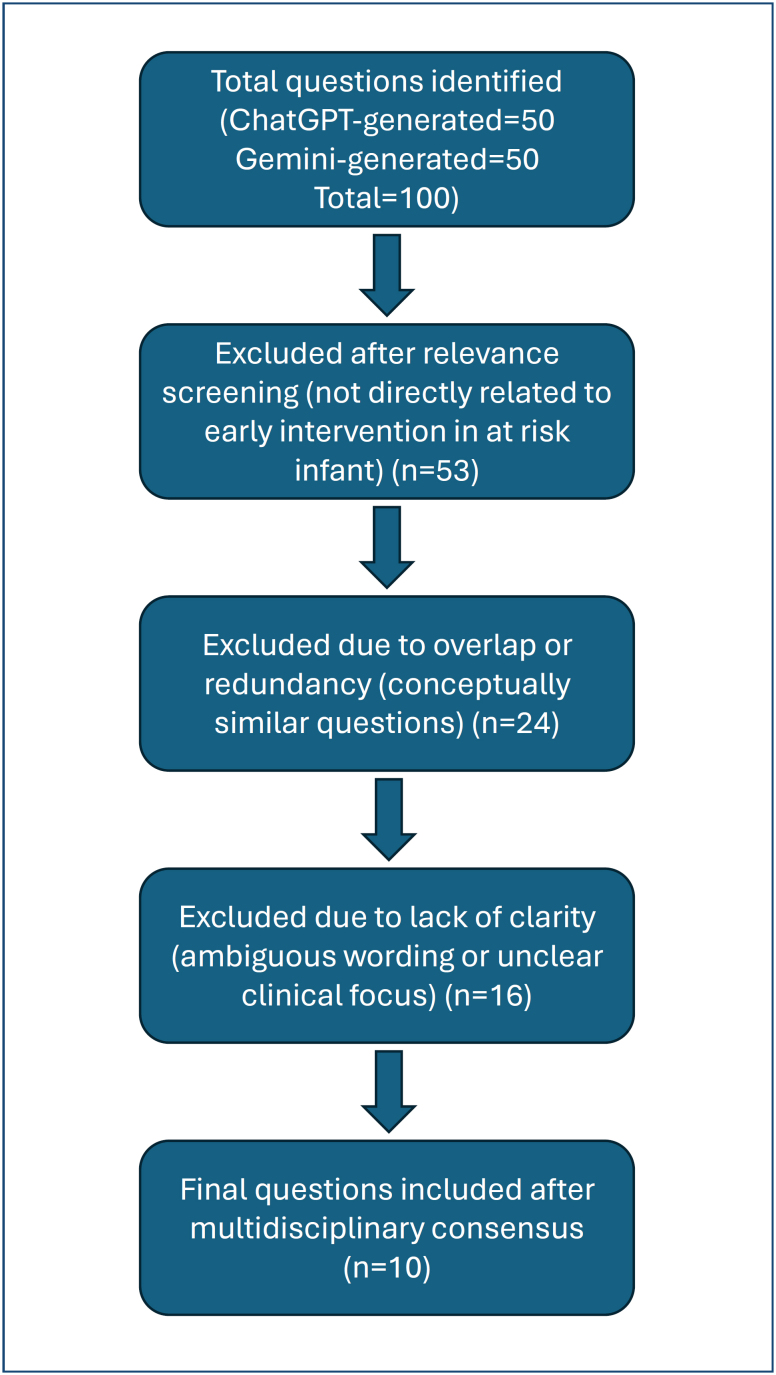
Flowchart illustrating the selection process of the final 10 questions.

From the initial pool of 100 questions (50 generated by ChatGPT and 50 by Gemini), a final set of 10 questions was selected through a structured consensus process conducted by three researchers (a child development specialist, a physiotherapist, and a midwife). The selection process consisted of the following steps: (a) Relevance screening: Questions not directly related to early intervention in at-risk infants were excluded. (b) Overlap and redundancy assessment: Conceptually similar or duplicate questions were merged or eliminated. (c) Clarity and comprehensibility: Questions with ambiguous wording or unclear clinical intent were excluded. (d) Multidisciplinary relevance: Priority was given to questions addressing medical, developmental, and rehabilitative aspects of early intervention. All three researchers independently reviewed the questions and subsequently met to reach consensus through discussion. Only questions approved unanimously were included in the final analysis (Figure 1 and Table 1).

**Table 1:** Questions.

No	Question
1	What exactly is early intervention?
2	How long will the early intervention program last?
3	Which therapies will be used in the early intervention program?
4	In which cases is early intervention recommended?
5	Who is a member of the early intervention team?
6	How often should we go to therapy?
7	What arrangements should we make for early intervention at home?
8	Is early intervention always successful?
9	What should we do if we don't get results?
10	What are the consequences of late initiation of early intervention?

### ChatGPT and Gemini usage

The questions were sent using ChatGPT version 4.0 and Gemini version 1.5. In order to prevent the applications from being affected by past searches, the browser history was cleared first, then a new ChatGPT and Gemini account was opened for the first time. Then, 10 questions determined by the researchers were asked in order. For each question, only a single, isolated prompt was used, and the first response generated by the applications was recorded without follow-up queries or iterative refinement. This approach was intentionally chosen to standardize comparisons; however, it does not reflect real-world user behavior, where individuals often engage in iterative questioning or prompt refinement when seeking health-related information.

### Assessment

The quality of the responses was assessed by 10 independent investigators (2 special education specialists, 2 child development specialists, 2 physiotherapists, 2 midwives, and 2 pediatricians) using the four-grade system described by Mika et al.^17^. The scale is scored from 1 to 4. One means a perfect answer, and four means an inadequate answer (Supplementary Table 1).

The readability of the apps’ answers was assessed using WordCalc software, where the answers to each question were pasted into a readability calculator and the corresponding Flesch-Kincaid Grade Level was recorded. The evaluators assessed the answers of both ChatGPT and Gemini apps, blind to the app that provided the answers.

### Statistic

The software package Statistical Package for the Social Sciences Statistics for Windows, Version 29.0 (IBM Corp., Armonk, NY, USA) was used to analyze the data. The results of the quality assessment of the answers are given as median and interquartile range (IQR). The Wilcoxon signed-rank test was used to compare paired quality scores between ChatGPT and Gemini for each question, as the data were ordinal in nature and derived from a four-point rating scale. This non-parametric test was considered appropriate because the assumptions of normality cannot be met for ordinal scale data.

## RESULTS

The comparison of quality scores between ChatGPT and Gemini is presented in Table 2. ChatGPT provided significantly higher-quality responses for Question 4 (p=0.025), whereas Gemini provided higher-quality responses for Question 7 (p=0.033). No statistically significant differences were observed for the remaining questions (Table 2).

**Table 2:** Comparison of the answers given by the applications.

No	Quality of answer				z	p
	ChatGPT		Gemini			
	Median	IQR	Median	IQR		
1	1.50	1.25	2.00	0.25	0.447	0.655
2	2.00	1.25	2.00	1.00	1.667	0.096
3	2.00	1.25	2.00	1.25	0.477	0.655
4	1.50	1.00	2.00	0.50	2.236	**0.025** *
5	1.00	1.00	2.00	1.25	1.414	0.157
6	1.50	1.25	1.50	1.00	0.272	0.785
7	2.00	3.25	1.00	1.00	2.126	**0.033** *
8	1.00	1.00	1.00	1.00	0.000	>0.999
9	1.00	1.00	2.00	1.00	1.732	0.083
10	2.00	0.00	2.00	0.25	1.000	0.317

IQR: interquartile range; Z: Wilcoxon signed test.

* p<0.05. Bold values indicate statistically significant results.

ChatGPT demonstrated lower Flesch-Kincaid Grade Level scores than Gemini for eight out of ten questions, while Gemini demonstrated lower scores for two questions (Questions 1 and 4). Detailed readability scores for each question are presented in Table 3.

**Table 3:** Flesch-Kincaid Degree Level of questions.

Question	ChatGPT	Gemini
1	11.2	10
2	9.8	12
3	11.9	12
4	12	11.8
5	9.2	12.2
6	11.3	11.9
7	9.1	9.5
8	11.9	12
9	10.8	11
10	11.3	12.1

## DISCUSSION

In this study, we evaluated the quality and readability of the ChatGPT version 4.0 and the Gemini version 1.5 application's responses about early intervention from families with at-risk infants. Our results showed that the responses of ChatGPT version 4.0 and the Gemini 1.5 app had similar quality. The observation that one model outperformed the other on isolated questions should be interpreted cautiously. Given that statistically significant differences were observed for only two out of ten questions, these findings may reflect question-specific variation or inherent differences in model training rather than systematic superiority of one model over the other.

Gemini was reported to be more scientifically interesting and accurate in spelling than ChatGPT. Comparison of the answers given by the applications^18^. In the evaluation of the recommendations given by ChatGPT and Gemini applications, ChatGPT and Gemini showed similar performance in pediatric orthopedic conditions^19^, while for family education on retinopathy of prematurity, Gemini was reported to have better readability scores but lower reliability scores^20^. Among the ChatGPT (GPT-4), Google Bard (Gemini Pro), Microsoft Bing Chat, and Google SGE applications, ChatGPT showed an overall high performance in providing valuable and reliable information to carers of children with cancer^21^ and it was found that ChatGPT had higher readability^22^ than Gemini, but the quality of Gemini was higher. ChatGPT and Gemini both demonstrate impressive capabilities in text comprehension and generation, and each has distinct strengths in several areas^23^. Studies comparing ChatGPT and Gemini in different fields are available in the literature, but there are no studies evaluating the accuracy of ChatGPT and Gemini responses in mothers with high-risk infants. The results of the study are generally similar to the literature. Therefore, the quality differences observed in this study should not be interpreted as overall model dominance, but rather as context-dependent performance variations.

Differences observed between comparative studies of LLMs may be partly explained by the clinical domain in which the models are evaluated. In procedural and guideline-driven specialties such as orthopedics, higher-quality responses may be associated with structured, protocol-based knowledge, whereas in ophthalmology or neonatal care, the need for precise medical terminology may increase the perceived technicality and reading level of responses. In contrast, early intervention focuses heavily on family-centered communication, developmental guidance, and interdisciplinary coordination, which may favor more explanatory and accessible language rather than strictly technical detail. Taken together, variations across comparative studies likely arise from differences in clinical complexity, communication goals, and audience expectations rather than from consistent model-specific advantages.

## CONCLUSION

This study assessed the quality and readability of responses provided by ChatGPT version 4.0 and GEMİNİ application to questions related to early intervention for individuals with at-risk infants. The findings showed that both ChatGPT and Gemini produced responses of similar quality, however, Gemini generally required a higher reading level than ChatGPT.

While both applications provided acceptable answers, in terms of readability, ChatGPT responses tended to demonstrate lower reading grade levels compared to Gemini, suggesting potentially greater accessibility for general users. However, the observed differences were modest and varied across individual questions. Future research should broaden the scope by including different user demographics and investigating the accuracy of responses on other healthcare topics. Furthermore, integrating user feedback can enhance the personalized utility and practical application of these AI systems in healthcare communication. Future studies should incorporate iterative prompt strategies to better approximate real-world user–AI interactions in health information seeking.

### Limitations

This study is the first to compare the quality and readability of responses of two advanced AI systems, ChatGPT-4.0 and Gemini, in the context of early intervention for at-risk infants. This study provides novel insights into the performance of LLMs in early intervention contexts. However, this study had some limitations. A major limitation of this study is that the initial question pool was generated by LLMs rather than derived from empirically collected parental queries. Consequently, the selected questions represent the models’ perception of common concerns rather than actual question frequencies among parents. This ­methodological choice may introduce bias and limit the generalizability of the findings to real-world parental information-seeking behavior. Another important limitation of this study is the use of single, non-iterative prompts for each question. In real-world settings, users frequently refine their questions, request clarifications, or ask follow-up questions when interacting with AI-based systems. The absence of iterative prompting in this study may limit the generalizability of the findings, as the quality and readability of AI-generated responses may improve or change with prompt engineering or continued interaction. The study only analyzed a specific set of questions related to early intervention for at-risk infants. A larger pool of questions could be examined to generalize the findings. Another limitation is that the study evaluated AI responses in a controlled environment and did not include real user feedback.

## Data Availability

The datasets generated and/or analyzed during the current study are available from the corresponding author upon reasonable request.
